# An Overview of Conventional and Emerging Analytical Methods for the Determination of Mycotoxins

**DOI:** 10.3390/ijms10010062

**Published:** 2009-01-02

**Authors:** Irena Kralj Cigić, Helena Prosen

**Affiliations:** University of Ljubljana, Faculty of Chemistry and Chemical Technology, Aškerčeva 5, SI-1000 Ljubljana, Slovenia. E-Mail: irena.kralj-cigic@fkkt.uni-lj.si

**Keywords:** Mycotoxins, analysis, sample preparation, screening, food, animal feed, method validation, reference materials

## Abstract

Mycotoxins are a group of compounds produced by various fungi and excreted into the matrices on which they grow, often food intended for human consumption or animal feed. The high toxicity and carcinogenicity of these compounds and their ability to cause various pathological conditions has led to widespread screening of foods and feeds potentially polluted with them. Maximum permissible levels in different matrices have also been established for some toxins. As these are quite low, analytical methods for determination of mycotoxins have to be both sensitive and specific. In addition, an appropriate sample preparation and pre-concentration method is needed to isolate analytes from rather complicated samples. In this article, an overview of methods for analysis and sample preparation published in the last ten years is given for the most often encountered mycotoxins in different samples, mainly in food. Special emphasis is on liquid chromatography with fluorescence and mass spectrometric detection, while in the field of sample preparation various solid-phase extraction approaches are discussed. However, an overview of other analytical and sample preparation methods less often used is also given. Finally, different matrices where mycotoxins have to be determined are discussed with the emphasis on their specific characteristics important for the analysis (human food and beverages, animal feed, biological samples, environmental samples). Various issues important for accurate qualitative and quantitative analyses are critically discussed: sampling and choice of representative sample, sample preparation and possible bias associated with it, specificity of the analytical method and critical evaluation of results.

## 1. Introduction

Awareness of mycotoxins has grown mainly in the last fifty years since the discovery of aflatoxins in the 1960s, although they have accompanied mankind from the very beginnings and were probably associated with several mysterious diseases known from history [1]. Mycotoxins are now recognized as prevalently toxic compounds produced as secondary metabolites by various fungi and excreted into their substrates. These substrates frequently include plants grown and stored for human or animal consumption as well as processed food. Mycotoxins and the associated health disorders in humans and animals have been recognized as a major health and economical problem [1, 2], which dictates measures to minimize the exposure by applying proper agricultural practice, storage of products and control of the products intended for human or animal consumption [3–10]. Moreover, there is also a growing awareness of the mycotoxin presence in the living and working environment and associated disease [11–14]. The control measures to ensure mycotoxin-free food, feed and environment imply chemical analysis of these contaminants in a great variety of samples, further complicated by the structural diversity of mycotoxins which call for different analytical methods.

The aim of the present review is to present the state-of-the-art of the conventional and emerging analytical and sample preparation methods for the most important mycotoxins in various matrices where they may be encountered. Besides, screening methods are also covered. Special emphasis is on the pitfalls of the mycotoxin analysis and the errors that may be inherent or introduced in each step of the analytical method. As this is a very broad scope, the review covers only the methods and techniques published in the last decade.

### 1.1. Diversity and impact of mycotoxins

Various commonly found types of molds produce and excrete mycotoxins. Between 300 and 400 different mycotoxins are known today, but not all of them are present in higher concentrations or have a significant health or economical impact and therefore have to be determined in various matrices [15, 16].

**Figure 1.**
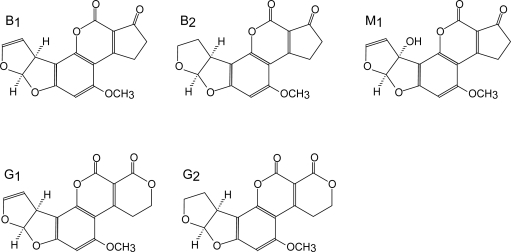
Structures of aflatoxins {AFs} B1, B2, M1, G1, G2.

**Figure 2.**
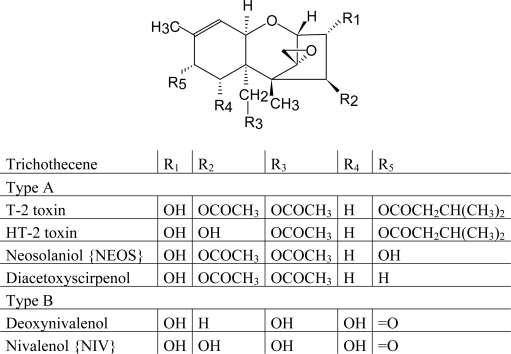
Structures of some important trichothecenes.

**Figure 3.**
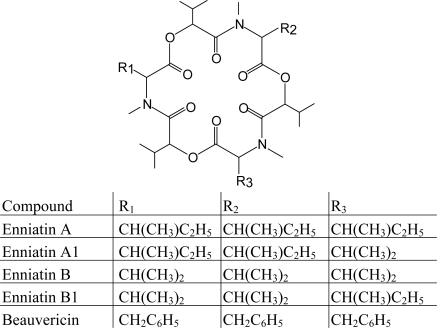
Structures of enniatins.

**Figure 4.**
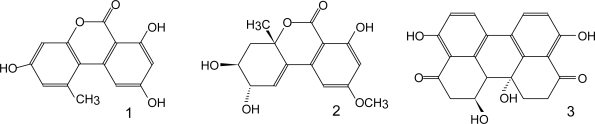
Structures of some *Alternaria* mycotoxins: alternariol {AOH} (1), altenuene {ALT}(2), altertoxin I{ATX-I}(3).

**Figure 5:**
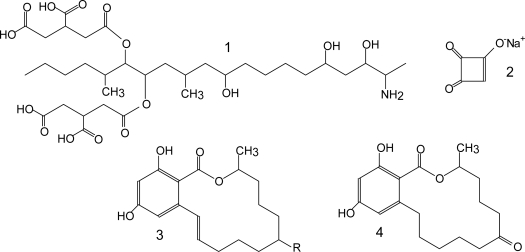
Structures of some Fusarium mycotoxins: FUM B_1_ (1), moniliformin (usually as sodium or potassium salt) (2), zearalenone {ZON} (R =O) and zearalenol {ZOL} (R -OH) (3), possible metabolite zearalanone {ZAN} (4).

**Figure 6.**
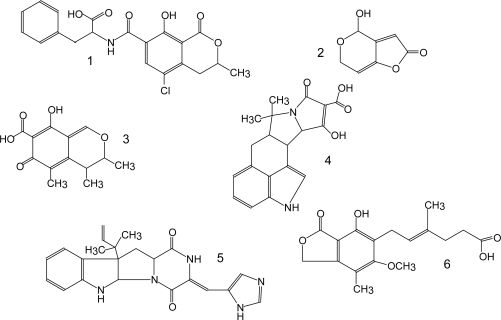
Structures of some important mycotoxins produced by *Penicillium* and *Aspergillus* species: ochratoxin A {OTA}(1), patulin {PAT}(2), citrinin {CIT} (3), cyclopiazonic acid {CPA} (4), roquefortin C {RQ} (5), mycophenolic acid {MPA}(6).

Table 1 lists the major groups of mycotoxins or individual compounds that are the most interesting from the analytical point of view as stated above. The abbrevations in the Table 1 are used throughout the text. Moreover, fungal species that produce them and the most common health disorders they cause are also listed. The chemical structures of these compounds are shown in Figures 1–6. There are other toxicologically important mycotoxins not included in Table 1 as they are less frequently encountered, e.g. penitrems, thomitrems, lolitrem, verruculogen, griseofulvin, chaetoglobosin, sambutoxin, citreoviridin, apicidin, roridin, monacolin K, phomopsins, sporidesmins, AAL toxins, satratoxins etc. [1, 2, 12, 17].

The health disorders listed in Table 1 are those most frequently associated with the exposure, but especially the data on acute toxicity of some mycotoxins show considerable variation in the available studies [1, 2, 7, 16, 18]. The susceptibility of animals (and humans) varies with species, age, nutrition, length of exposure and other factors as well [1, 2]. Evaluation of adverse health effects is complicated by co-existence of various mycotoxins in food and feed and their possible synergistic action [16, 19]. Many mycotoxins exhibit a rather non-specific action at the usual exposure levels, e.g. immunosuppression [2] and consequently can increase susceptibility to other illnesses [7]. Although it has been postulated that several unexplained disease outbreaks from the past were caused by the consumption of mycotoxin-contaminated food [1, 2, 7, 20], e.g. Balkan endemic nephropathy [19, 21], other environmental factors such as drinking water contamination may also play a role in their occurrence [22]. At present, there is no firm evidence that the normal daily exposure to some mycotoxins listed in Table 1 (RQ, MPA) would lead to health disorders [23, 24] or these occur only at higher intake levels (e.g. for PAT) [25], but nevertheless the maximum tolerable daily intake and residual levels in foods have been established [1, 25].

### 1.2. Diversity of samples containing mycotoxins

Molds and their metabolites are present everywhere in the environment, which implies a wide variety of samples that might contain mycotoxins and have to be analyzed. Table 2 lists the samples an analytical chemist will most frequently encounter. The detailed analytical approaches performed for these samples, however, are discussed in Section 5 of this review.

Because of the deleterious health effects of mycotoxins, their levels have been strictly regulated especially in food and feed samples. While European Union has in general set somewhat lower allowable residue levels than US Food and Drug Administration, they are usually in the μg/kg–mg/kg range for the majority of compounds [1, 4]. Various types of grains are the most burdened in terms of different possible compounds, mostly occurring pre-harvest and some also post-harvest during storage, although the latter is easier to control [1, 2, 4, 20, 26]. The first evaluation of food and feed is by inspection for the visible fungal contamination as well as for the characteristic moldy odor, although these factors don’t necessarily correlate with the mycotoxin concentration and are also not applicable to processed foods.

In samples of animal origin, such as meat, milk, dairy products and eggs, mostly aflatoxin metabolite M1 is expected and also OTA in some tissues (liver, kidney). Other mycotoxins haven’t been shown to exhibit carry-over from feed to animal products [1, 3]. Cheese deliberately inoculated with presumably non-toxicogenic molds may in addition contain MPA, RQ [23, 24] and to a certain extent also CIT and CPA are expected [19, 27]. Fish may contain RALs [28]. Special care has to be taken while evaluating mycotoxin content in processed foods, as during processing, especially heating, the content of mycotoxins is generally reduced, but degradation products of both higher or lower toxicity may form [9, 19].

Another group of samples in Table 2 are from the indoor environment. They have been gaining in importance since the recognition of the “sick building syndrome”, a chronic illness associated with fatigue, allergic reactions, neurological symptoms and higher incidence of cancer. Affected people live in mold-infected buildings with fungal spores containing mycotoxins present in the air and house dust [11, 12], but can be exposed also in the working environment [13, 14]. Analysis of human samples (blood, urine, milk) is therefore also important to evaluate the extent of exposure from environment and food [29, 30].

Finally, environmental samples such as soil and environmental waters may provide information on the fate of mycotoxins formed in fungally infected agricultural crops [31, 32] or of anthropogenic origin [33].

## 2. Screening methods

Different types of samples and their vast number, as well as chemical diversity of mycotoxins and their simultaneous occurrence in samples present a need for rapid multi-analyte methods suited for various matrices. Moreover, they should be sensitive enough to detect mycotoxins below the legally imposed limits. Separation methods coupled to sensitive and/or selective detection methods fulfill these requirements and are at this time the most definitive, but their drawback is that they are preceded by time-consuming sample extraction and clean-up, except if the raw extracts are analyzed (e.g. with LC-MS/MS). They are also rather expensive and demand specially trained personnel. For these reasons, the traditional as well as novel screening methods have found a wide application area in mycotoxin analysis [34]. They usually provide rapid and sensitive detection, are very cost-effective and easy to use, thus they can be used by non-specialists and under field conditions as well. Although there is a greater emphasis on selectivity in novel assays [34], the main drawback of many screening methods is still their cross-reactivity. Positive results should therefore be confirmed with more selective methods to avoid misinterpretations [35].

### 2.1. Immunoassay-based methods

These methods are based on the interactions between antibodies and antigens, which are in this case mycotoxins. Antibodies are highly specific for structurally very different compounds, but can show considerable cross-reactivity for structural analogs, as they recognize only specific chemical groups - the epitope [15, 36]. Mycotoxins are small, non-immunogenic molecules (haptens) and have to be bound to suitable carrier proteins to elicit adequate immune response and production of antibodies in an animal [37]. Novel methods of recombinant antibody production can circumvent the binding of hapten to protein and inherent risk that the free hapten would not be detected in samples, as well as animal immunization [38]. Another compound, variously named marker, tracer or label, is involved in the assay to facilitate detection. The marker may be radioactive in radioimmunoassay (RIA) - rarely used now, or a chromogenic or fluorogenic compound reacting with enzyme in enzyme immunoassay (EIA, ELISA) or in fluorescence immunoassay (FIA), respectively.

ELISA (enzyme-linked immunosorbent assay) is the most frequently applied type of assay [39, 40]. Substrate for the enzyme is usually a chromogenic substance. The assay is mostly performed in a 96-well plate, allowing simultaneous analysis of up to 45 samples in duplicate. Incubation times are 0.5–2 h and the developed color is usually measured spectrophotometrically [41]. Other types of detection are possible, e.g. amperometric [42] or by differential pulse voltammetry [43]. The most simple, however, is the visual comparison of color intensity, providing either semiquantitative results [44] or a yes/no response at a certain concentration level [34] or concentration range [41].

Other immunoassay type employs fluorescent markers, which involve fluorogenic substrate reacting with enzyme-linked analyte [34, 40, 45, 46] or measure fluorescence polarization induced by increased molecular mass of antibody-bound labeled mycotoxin [34, 39, 40, 41, 47].

Marker-free immunoassays are based on natural fluorescence of some mycotoxins (AFs, OTs), conductometric or impedimetric measurements [34]. Surface-plasmon resonance-based immunoassays (SPR) employ the change of surface optical characteristics upon binding of antibodies to the surface-bound antigen. The amount of free antibodies correlates with the mycotoxin content of the sample with which they are incubated [34, 39, 41, 48, 49].

Besides the various detection modes, different constructions have also been developed in the field of immunoassay methods. The most common design is a multi-well microtiter plate used in classic ELISA with spectrophotometric or electrochemical detection [50]. Flow-through or flow-injection immunoassays use immobilized proteins to separate antigen-antibody complexes and detect only the free marker [40, 42, 45]. Mycotoxin-tagged liposomes have been prepared to develop a flow-injection liposome immunoanalysis (FILIA) [51]. Flow-through test may be used as another name for immunofiltration, where antibodies are immobilized on a semi-permeable membrane. Such devices are simple to use in field conditions with visual detection, but can be also quantitatively evaluated with instrumental detection in laboratory settings [41, 44]. Finally, various immunostrips or immunodipsticks, also named lateral flow devices (tests), have been developed for mycotoxin testing in the field, using either membrane with immobilized antibodies or dry reagents on the test strip. In the latter case, the sample would reach and dissolve the reagents by lateral capillary flow and the test is thus self-developing [34, 39–41].

With respect to mycotoxin types, various innovative immunoassays mentioned above were developed for most of the main groups: AFs [34, 42–44, 47, 50], trichothecenes [34, 41], FUMs [34, 38, 48, 51], RALs [34, 45], OTA [34, 37] and PAT [46]. On the other hand, immunosensor arrays, designed to detect several mycotoxins from different groups simultaneously, are gaining in importance as well [34, 41, 49, 52–54].

Immunoassays with colorimetric and fluorimetric detection generally display favorable detection limits (LOD 1–20 μg/kg) [34, 40, 55], but it must be re-emphasized that they should be used as a screening tool only to detect possible contamination with mycotoxins. It has been established that while false-negatives are rare, false-positives are more frequent and depend on many factors. Besides the inherent cross-reactivity, other conditions influence the result: temperature, sample viscosity, pH, ionic strength [41]. If no sample clean-up or extraction is performed before the test, matrix effects might be expected [39, 55], sometimes leading to significant overestimation of mycotoxin concentration [39, 56]. Extraction usually involves polar organic solvents, although they are suboptimal for most mycotoxins. Typically methanol is used, which is tolerated to a various percentage depending on the design of the immunoassay [54]. Co-extracted substances, e.g. fat, may also cause problems. For water-soluble mycotoxins, water or buffers can be used instead [41], but in any case, additional clean-up may be needed, especially for colored samples [57]. In case of visual estimation of tests, a concentration range should be considered rather than a cut-off value which is difficult to establish due to the analog nature of both color development and human vision [41]. For all the reasons listed above, samples shown to be positive on mycotoxins should be re-analyzed by the reference method.

### 2.2. Sensors and biosensors

Sensors are devices composed of two elements: the molecule (usually biological), which selectively reacts with the compound(s) of interest (recognition element) and is in contact with a transducing element converting the change of the physical variable produced by the reaction into a measurable signal. In this respect, biosensors are those using biological molecules/entities as recognition elements: antibodies, enzymes, bacteria, receptors, DNA [34, 58, 59]. Transducing elements are usually optical or electrochemical.

Some of the immunoassay approaches described in the previous section can be considered as biosensors, provided that the measured signal originates directly from the antibody-antigen complex in contact with the transducing element [34]. The latter can be an optical detector based on surface plasmon resonance [40, 48, 49, 60], fluorescence [61], optical waveguide lightmode spectroscopy - OWLS [53] or total internal reflection ellipsometry - TIRE [34]. Another possibility is an electrochemical detector based on potentiometry with carbon working electrode [50, 62], differential pulse voltammetry, conductometry [34], chronoamperometry [63] and electrochemical impedance spectroscopy [64]. Some of these sensors are for single mycotoxins and others are made for multi-toxin sensing (bisensor array). Other types of biosensors include enzymatically-mediated electrochemical sensors based on potentiometry, and DNA sensors [34, 59].

The advantages and disadvantages of biosensors are essentially the same as those of immunoassays: they are cheap, fast, portable, very suitable for routine screening of samples, but they suffer from the selectivity and reproducibility problems. The results should be therefore confirmed with the reference method [34, 58]. However, some electrochemical biosensors show excellent sensitivity with LODs below 0.1 μg/kg [34, 50, 63], while those based on optical detection achieve LODs comparable with ELISA (0.5–10 μg/kg) [34, 53].

Another type of sensors that mimic the biochemical recognition molecules is the one based on molecularly imprinted polymers (MIPs) [34, 40, 65–68]. These functional materials have found a wider application in the field of selective sample preparation and will be discussed in more detail in the next section. MIP-based sensors have some advantages over biosensors: better stability in organic solvents, decreased degradation and loss of active sites [34, 65], but so far only the sensors with fluorescent tracers displaced by the analyte have been designed [67]. Problems of these sensors are connected with problems of MIP materials in general: selectivity [34, 67], reproducible preparation [68], sample contamination [66, 68]. Nevertheless, MIP-based sensors are very promising and are expected to find wider application in the future.

### 2.3. Other direct screening methods

Several approaches making use of the different chemical and physical characteristics of mycotoxins will be mentioned here: some already established, others just emerging. The best known method of the former is thin-layer chromatography (TLC), which, although used also for the more accurate analyses, can be applied for the screening purposes as well with visual or densitometric detection. Compared to ELISA, it shows better repeatability and is less prone to overestimation of the concentration due to its better selectivity, but requires more extensive sample clean-up and is more time-consuming [69].

Flow-injection systems use a flow-through detector with rather high specificity for the analytes of interest. Examples are the system for total AFs using fluorimetric screening [70] and system for RALs using electrochemical detection [71]. In both cases an extraction and clean-up step is required. In case of samples with total concentration close to or above threshold limit, confirmation analysis is needed with a more selective method [70, 71]. Static fluorimetric analysis of previously extracted and derivatized analyte was applied for DON screening in grains [72].

An interesting AFB detection method is based on acetylcholinesterase inhibition by AFB1, subsequent enzyme substrate addition and spectrophotometric measurement of the product. The assay is very fast and quite sensitive, but strongly depends on the temperature and methanol concentration. It also shows a significant cross-reactivity to other AFs [73]. Another bioassay for trichothecenes was developed, exploiting the inhibition of enzyme β-galactosidase in the yeast with subsequent colorimetric detection of the product of enzymatic reaction. With the additional use of the toxicity-enhancing agents, LODs comparable with ELISA were achieved [74].

### 2.4. Indirect screening methods

These methods are based on the measurement/determination and interpretation of a parameter or compound(s) in the sample that change relative to the mycotoxin concentration. The relation has to be established on a series of samples analyzed by a reference method. Most of these methods are also non-invasive, meaning less of the sample manipulation and faster throughput.

Various methods using infrared spectroscopy have been developed, e.g. Fourier transform mid-infrared spectroscopy (FTIR) in diffuse reflection (DR) [75] or attenuated total reflection (ATR) mode for determination of DON in wheat or maize [39, 75, 76]. Principal component analysis (PCA) and cluster analysis were applied to evaluate the spectra and classify them on the basis of the mycotoxin contamination [75–77]. Spectra obtained in ATR mode were superior to DR spectra in terms of classification efficiency, e.g. samples with DON concentration 2.5–12.1 mg/kg were in 100 % classified as contaminated [75]. FTIR-ATR measurements with partial least squares (PLS) as a regression method were successfully applied to evaluate OTA contamination in dried grapes [78, 79]. Near-infrared spectroscopy (NIR) in the transmission mode with PCA and PLS calculations were used to develop a screening method for DON in wheat [39, 80]. NIR in reflectance mode and modified PLS algorithm were applied to determine total AFs, AFB1 and OTA in red paprika [81]. All of the above methods are applied without any sample preparation except milling and sieving [77], meaning great savings in time, but the disadvantage is that they are valid only for the sample matrix used during calibration [39]. Even if this condition is met, poor spectral reproducibility may still occur for samples of the same type, but from different regions [78].

A quite different approach for indirect detection of mycotoxins in sample is detection of volatile metabolites of the fungi. In this respect, a differentiation should be possible between toxicogenic (mycotoxin-producing) and non-toxicogenic strains. Determination of volatiles produced by fungi may be performed with the established methods, e.g. GC-MS with previous headspace extraction [82, 83], but there is an increasing number of publications where the development of an electronic nose (e-nose) for this purpose is described. E-nose is an array of gas sensors based mostly on metal oxides. Since the response is non-specific, it has to be calibrated against reference method for volatiles and processed by chemometric methods to be able to classify subsequent samples in the appropriate groups [84–88]. So far, this approach has been successfully used for the determination and even prediction of concentration of OTA (classification limit 5 μg/kg) and DON in barley [87] and PAT in apples [86]. Other publications report only detection of mold growth on wheat [84, 85], the approach that can be as effective as sensory panels [88]. However, it is possible to differentiate between toxicogenic and non-toxicogenic strains of fungi by detecting specific volatile metabolite(s), albeit as far as now only by GC-MS [82, 83]. Again, sample preparation step is shortened or even absent in the case of e-nose use, but matrix dependence is significant in this approach as well.

As an estimation of the total fungal biomass in samples, e.g. maize, the determination of fungal metabolite ergosterol is often performed with HPLC [76, 89, 90].

## 3. Sample preparation and pre-concentration

One of the crucial steps for the qualitative and quantitative determination of individual mycotoxins is the sample preparation and pre-concentration. Several different extraction procedures are used for the purpose and will be overviewed in this section. Some of these are well-established techniques and others just emerging, which have yet to prove their relative advantages and demonstrate shortcomings. The emphasis in the field of sample preparation in the last few decades has been on the minimization of solvent use, especially of environmentally and health-harmful chlorinated solvents, as well as on the miniaturization of the procedures, meaning also smaller sample size, while at the same time maintaining the efficiency. The other field of development is in the highly analyte-specific extraction techniques with added benefit of reduced need for subsequent purification of the extracts. On the other hand, older sample preparation techniques, although usually time-consuming and more labor-intensive, have the advantage of long-term use: the possible pitfalls are well recognized and can thus be relatively easily avoided. One should bear in mind that several serious systematic errors are possible in this step of analysis, some of which will be mentioned shortly, and then again overviewed in the Section 6 of this review.

### 3.1. Solvent extraction

The oldest, but still frequently used sample preparation technique is solvent extraction (SE), in the case of liquid samples also named liquid-liquid extraction (LLE). Both aqueous - e.g. buffers, hot water, and organic solvents - e.g. acetonitrile, chloroform, methanol, ethyl acetate etc. are used. Samples may be solid or liquid. With liquid samples this step serves as an enrichment and/or clean-up procedure, while with solid samples SE is a necessary pre-step for further manipulation of sample extract. Different modes of SE can be employed, such as conventional Soxhlet extraction, used for solid samples [91] and ultrasonic extraction [92]. One well-recognized drawback of Soxhlet extraction is the possible production of artifacts from thermally less stable compounds, although it hasn’t yet been shown for mycotoxins. More recent techniques include supercritical fluid extraction (SFE) [71], pressurized liquid extraction (PLE), also known as accelerated solvent extraction (ASE) [45, 93–96] or microwave-assisted extraction (MAE) [97]. They require less solvent and usually a better extraction efficiency (in terms of extraction yield or recovery) is reached comparable to classical SE/LLE. Furthermore, by means of modern MAE and especially SFE approaches, both extraction and clean-up can be performed in a single step [97, 98]. One drawback of PLE/ASE is seen for complex samples, where rather dirty extracts are obtained because of matrix coextraction [91].

### 3.2. Solid-phase extraction

Solid phase extraction (SPE) is another possibility for direct extraction of liquid samples, in mycotoxin analysis most often used for clean-up and pre-concentration of extracts. Originally, SPE has been performed on broad-range, non-specific stationary phases (reverse-phase, normal-phase, ion-exchange, activated carbon etc.), while recently there has been greater emphasis on the use of another type of materials, which enable very selective binding of target molecules and sometimes also higher recoveries [99]. The most popular are immunoaffinity (IA) materials, while molecularly imprinted polymers (MIP) are an emerging, cheaper and very promising alternative [65, 100].

SPE on non-specific materials is still often employed in mycotoxin analysis. This kind of extraction is mainly performed for multiresidual determination of mycotoxins followed by LC-MS/MS [101, 102], where greater selectivity of SPE material would be a limiting element.

IA materials are prepared by binding antibodies specific for a given mycotoxin to a specially activated solid-phase support. Immunoaffinity extraction (IAE) is performed generally for all mycotoxins in very diverse matrices [98, 103–111]. More complex samples require combination of different clean-up procedures (for example LLE, SPE, precipitation of matrix substances) with immunoaffinity columns (IAC) [109, 112]. The IAC are not absolutely selective for individual mycotoxins as also mycotoxin analogues are usually bound to the material. However, when a separation technique is used for the subsequent determination, this does not present a problem [104, 108, 109]. As an additional advantage, IAC for different groups of mycotoxins are commercially available [103, 111]. The main problem concerning clean-up via immunoaffinity columns is the denaturation of the antibodies in the presence of even low concentrations of organic solvents, therefore expenses are rather high [106]. One way to overcome this problem is by extract dilution, although it may cause a loss of sensitivity. The capacity of IAC may also be a problem. Lately, sol-gel immunoaffinity columns have become available. Their use is less time-consuming and less costly; columns have superior storage stability [107].

Usually, the antibodies for IA materials are prepared *in vivo* by immunization of animals with mycotoxins bound to suitable carrier proteins, but lately IAE on synthetically generated antibodies with specific affinity to mycotoxins has been reported [38, 110].

Another modern SPE with specific material is moleculary imprinted solid phase extraction (MISPE), which enables extraction of a single mycotoxin or a whole group. MIPs are tailor-made synthetic materials with artificial binding sites, able to selectively recognize a target molecule [65, 100]. Preparation of moleculary imprinted polymers is based on polymerization of selected monomers around the template molecule. As a template, target molecule or the so called mimic template can be used, which is usually a structural analogue of the analyte [65, 113]. Preparation procedure of MIPs based on target molecule as the template is problematic with trace analysis, as there may remain residues of the template in the polymer material and therefore leaking of analyte is observed [65, 68]. Additionally, some mycotoxins are too toxic or too expensive for preparation of MIPs [68]. On the other hand, there are also difficulties with selecting the optimal mimic templates. Structurally optimal compounds are not always commercially available as their isolation or preparation could be expensive, especially in the amounts needed for MIPs preparation. Selection of suboptimal mimic template leads to a limited molecular recognition effect [68]. For this reason, many investigations on preparation of MIPs with mimic templates are published: for ZON [66, 67, 100], DON [114], OTA [68] and moniliformin [115]. Usage of MISPE as a clean-up method for the mycotoxin determination increases because of its efficiency in very complex matrices and because of MIPs stability, e.g. in apolar solvents, especially when compared with IA materials [68].

Conventional SPE materials are also used for the clean-up purposes. The most successful is often a combination of different materials [58]; one of such multifunctional clean-up columns is MycoSep [98, 116]. They contain a combination of different sorbents, e.g. charcoal, ion-exchange resins etc., and are designed for easy and rapid handling - extract is obtained within 30 s [39]. The major advantages of these columns are the reduced amount of solvent and avoidance of time-consuming rinsing steps required in single-phase SPE. Other interesting development in the SPE field is primarily the introduction of new materials, e.g. surfactant modified zeolites [117].

All described SPE materials are usually packed in the open cartridges for off-line applications, but also other modes of extraction media are reported: package in short HPLC columns for on-line applications, multiwell extraction plate for high throughput analysis [65, 113], monolithic CIM disks [105] etc. As the sample preparation is frequently the crucial step in the determination of mycotoxins, some on-line extraction procedures, requiring less manipulation of the sample, were shown to be better compared to the off-line approach [24, 58, 118]. Most SPE procedures include the following steps: column conditioning, sample loading, column washing and analyte elution.

Another mode of SPE for solid or semi-solid samples is matrix solid phase dispersion (MSPD), where the sample and sorbent material are mixed homogenously. This mixture is than packed in cartridge and afterwards elution is performed. MSPD on non-selective materials, e.g. silica, phenylsilica, alumina, C_8_ and C_18_ has been reported for the determination of AFs in peanuts [119], RALs in fish tissue [28] and trichothecenes in maize flour [120].

Development of powerful separation-identification analytical techniques such as GC-MS and HPLC-MS/MS, which are nowadays routinely used, simplified sample preparation by employing only one single step of LLE or SPE [119, 121–125], but as will be discussed in Section 4, remaining matrix compounds may cause certain problems even with these analytical techniques.

### 3.3. Other extraction techniques

As the alternative to SPE procedures use of solid phase microextraction (SPME) is reported. This technique employs fibres coated with different stationary phases on which analytes are sorbed from liquid (aqueous) or gaseous samples. Desorption can be performed thermally in combination with GC analysis or with different solvents followed by HPLC analysis. SPME sampling in combination with HPLC analyses was performed for the determination of OTA, CPA, MPA [22, 23, 27, 28, 126, 127]. Another application involving SPME for the mycotoxin determination is the detection of volatile fungal metabolites [82, 83, 85], where also stir bar sorptive extraction (SBSE) can be employed [82]. SBSE uses a coated magnetic stir bar to capture analytes. The extraction phase is mostly PDMS. SBSE has higher recoveries and higher capacity than SPME fibres and therefore additional applications in this field are expected.

### 3.4. Other sample preparation techniques

Rarely, other sample preparation methods are also used, such as immunofiltration for DON [128] and AFB1 determination [44], diphasic dialysis extraction for PAT determination [129], coacervative extraction for simultaneous extraction and concentration of OTA in wines [130], use of supported liquid membrane for OTA determination [131] and capillary fused silica columns as the traps for the extraction of T-2 toxin from aqueous solutions [132]. Only individual applications are reported so far, but some may find wider application in the future.

Clean-up of the samples has also been reported with the addition of specific chemicals, e. g. zinc acetate [133], hydrogen carbonate and PEG [112], lead hydroxyacetate [134], mostly to precipitate matrix substances, e.g. fatty acids [135]. The majority of these procedures were described for the determination of OTA in different matrices.

## 4. Analytical methods

The vast majority of chemical analytical methods applied for accurate, selective and sensitive mycotoxin determination in various samples come from the group of separation methods: chromatography, electrophoresis. One of them, high performance liquid chromatography (HPLC) with different detectors is frequently used both for routine analyses and as confirmatory method for novel or screening techniques [35, 45, 51, 58, 71, 81]. For some mycotoxins, e.g. trichothecenes, gas chromatography (GC) is the method more often used [76]. Except for direct mass spectrometric methods, all the other analytical methods used for mycotoxin determination are either immunoassay-based or otherwise fall in the category of direct or indirect screening methods. They were described in details within the Section 2 of the present review.

Similarly to other analytical steps in the mycotoxin determination, the field of separation methods used for this purpose has shown a tremendous development in the last ten years, which can be easily observed from the comparison of analytical reviews of that time [25, 69, 136–140] and in the last few years [16, 19, 39, 141–145]. The great majority of the older and novel methods have detection limits well below the corresponding maximum residue levels in samples [39, 136, 139, 142]. The driving force behind this development is therefore not so much the sensitivity, but the greater simplicity and higher throughput of novel methods. The detector of choice has become the mass spectrometer, preferably even tandem mass spectrometer [16, 141–143]. Judging from the number of publications, fluorimetric detector for HPLC is still very popular due to its sensitivity, selectivity, rather low price and ease of use, although derivatization is necessary for most mycotoxins. Other detectors for HPLC are also used, most notably UV-spectrometric. Good chromatographic separation of analytes is mandatory in combination with all detectors except MS, where some peak overlapping is allowed, therefore the chromatographic conditions will be described only in the second sub-section on LC-FL. As regards the use of other separation methods, GC is often applied for volatile mycotoxins, followed by electrophoretic methods, modern thin-layer chromatography and others [19, 39, 144, 145].

### 4.1. Liquid chromatography coupled to mass spectrometry (LC-MS)

Liquid chromatography hyphenated to mass spectrometry (LC-MS) or tandem mass spectrometry (LC-MS/MS) has in the last ten years advanced to the status of the reference and definitive method in the field of mycotoxin analysis. The reasons are, among others, the development of efficient electrospray (ESI) and atmospheric pressure chemical ionization (APCI) interfaces for LC-MS coupling, the developments in the field of mass analyzers, and the considerably greater simplicity of use and affordability of tandem mass spectrometers. Probably the greatest merit goes to the ESI and APCI. Before their development, the LC-MS analyses of mycotoxins were performed using thermospray and fast-atom bombardment interfaces, but with significant difficulties [136]. Modern LC-MS instruments with ESI or APCI enable ionization in both positive and negative mode, as well as switching between them in the same chromatographic run [16], which means the best possible detection conditions for all analytes.

Most newly developed LC-MS methods enable analysis of a whole group of similar mycotoxins. For A-trichothecenes, a method using LC-APCI(+)-MS and deuterated T-2 toxin as an internal standard (IS) was developed [146], while in another research with tandem MS [147], ESI in negative mode yielded better results for four B-trichothecenes than APCI in either mode. Electrospray with MS/MS was applied to the semi-quantitative analysis of non-macrocyclic and macrocyclic trichothecenes in samples from indoor environments [148]. Six B-trichothecenes and metabolites were determined by LC-APCI(−)-MS with dexamethasone as an internal standard [149]. Trichothecenes from groups A and B were analyzed using APCI and tandem MS without IS [150] or by ESI and MS/MS with deepoxydeoxynivalenol as IS [151].

Several LC-MS methods have been published for the determination of estrogenic *Fusarium* mycotoxins - RALs (zearalenone and its metabolites) [142] with a single mass analyzer [109, 140, 152, 153] or tandem MS [28, 91, 140, 154–156]. The ionization was possible both by APCI [28, 109, 152, 154] and ESI [28, 91, 153, 156]. Additional focus was drawn to the determination of the conjugated analytes in samples [157] and of zearalenone metabolites [158]. Matrix compounds can cause a considerable suppression of analyte ionization [28, 39], therefore the use of internal standards is recommended for quantification. A structural analogue zearalanone has been widely used for this purpose [150, 153, 154], although this can be a metabolite as well [155]. Therefore, deuterated internal standards are used as better, albeit more expensive alternative [91, 109, 156, 159]. Additional confusion in the determination of RAL mycotoxins, especially in animal samples, can arise from the presence of structurally related anabolic substance zeranol and its metabolites, which are also ionized by ESI and should be considered in the method [33, 102, 160, 161].

Fumonisins are another group of *Fusarium* mycotoxins occurring mostly in corn. LC-MS methods employed for their analysis [142] apply single analyzer [162, 163] or ESI(+) with tandem MS [164, 165]. Enniatins and beauvericin, produced by the same fungal genus, have also been determined by LC-MS/MS [142, 166]. Moniliformin, a low molecular weight and highly polar mycotoxin produced by *Fusarium* species, is efficiently ionized in the negative mode of ESI and APCI [121, 142, 167], but may have to be derivatized in order to achieve retention on reverse-phase LC columns [142]. Alternatively, ion-pairing can be used for this purpose [167]. Methods for the analysis of *Fusarium* mycotoxins from different chemical groups with LC-MS/MS have been published as well, requiring optimized ionization conditions and switching between positive and negative modes for every group [39, 168, 169]. Use of isotopically labeled IS is preferable [163].

Aflatoxins are mycotoxins produced by *Aspergillus* fungal species, occurring in various food and feed commodities. However, the published LC-MS methods are relatively uncommon, mainly because of well-established, reliable and sensitive LC methods with fluorescence detectors measuring the natural fluorescence of aflatoxins [142]. Compared to that, there are several LC-MS methods dealing with the precursor sterigmatocystin [142, 170–172]. ESI ionization is applied in most LC-MS methods for aflatoxins [119, 172–174], APCI much less [122], as it seems to be less efficient than the former, except in the case of aflatoxin precursor sterigmatocystin [142]. Atmospheric pressure photoionization (APPI) might be a better alternative to ESI than APCI [142]. Most studies report absence of matrix effects and ion suppression in ionization [142], while Cervino *et al.* [174] found matrix independent response for AFB2 and AFG2 and strong matrix dependence for other aflatoxins even while using deuterated internal standards, therefore matrix-matched calibration was recommended.

Ochratoxins, especially ochratoxin A (OTA), are another group of harmful mycotoxins for which, due to their natural fluorescence, several well-established and sensitive LC-FL methods exist. Therefore, LC-MS methods aren’t as frequently encountered as for *Fusarium* mycotoxins. The first methods were published at the beginning of this review’s timespan [138, 175], utilizing ESI ionization. The same interface has featured in most methods up to date, used either in positive [101, 176] or negative mode [127, 173], the former offering a more specific fragmentation pattern [142]. Use of APCI interface results in lower sensitivity due to extensive fragmentation of OTA [142]. There are reports of decreased ionization efficiency because of matrix effects [142], therefore the calibration step is crucial. Nevertheless, some authors report excellent results with external calibration [101, 173], while in other studies ochratoxin B (OTB) was used as an internal standard [127, 142, 177]. Instrument sensitivity for OTB is significantly lower compared to OTA, therefore high concentrations of OTB are needed [127]. Although uncommon by now, the use of deuterated internal standards is preferable [176]. LC-MS has been comparatively more often used for the elucidation of OTA metabolites [142] and products of photodegradation [118].

Published LC-MS methods for the determination of less ubiquitous mycotoxins are not frequently encountered. *Alternaria* toxins in various fruit samples were determined by LC-MS/MS. ESI in negative mode offered the best sensitivity and specificity compared to ESI(+) and APCI in either mode [178]. Analysis of patulin in apple juice was successfully accomplished with a single mass analyzer in SIM mode [179]. Use of isotopically-labeled internal standard is advisable [180]. In another study, Takino *et al.* compared APCI and APPI for the same purpose and found that APPI provides lower chemical noise and ionization suppression even in the absence of internal calibration [181]. On the other hand, some authors found LC-MS considerably less sensitive than GC-MS and recommended it for structural confirmation only in spite of the more simple sample preparation [142]. Citrinin can be efficiently ionized by ESI and determined by tandem MS [172]. The most important ergot alkaloids and their isomers of lower toxicity have been determined by ESI-MS/MS [182]. Finally, there is a heterogenous group of mycotoxins produced mainly by *Penicillium* species, among them cyclopiazonic acid (CPA), mycophenolic acid (MPA) and roquefortin C. A method using LC-ESI-MS/MS in SRM mode was employed for CPA in milk samples [183]. Several methods exist for the determination of MPA, which is also a metabolite of an immunosuppression drug [142]. A full-scan and SRM LC-MS/MS method for the analysis of six *Penicillium* mycotoxins with low detection limits was developed as well [17].

The strength of LC-MS methods lies in the possibility to perform multi-analyte analyses. Due to the highly specific mode of detection in tandem MS (SRM), chromatographic separation is slightly less important and overlapping peaks can be tolerated. Still, the challenges of achieving right chromatographic conditions remain especially in modulating pH and additives in the mobile phase to promote the optimal ionization of analytes in the ion source. Since mycotoxins vary greatly in the polarity, molecular mass etc., optimal ionization can only be accomplished in modern instruments with rapid switching between negative and positive ionization modes required by different chemical classes [136]. Sample preparation and especially extract purification is problematic as it relies heavily on the polarity and functional groups of analytes; simultaneous extraction of different chemical groups without significant matrix carry-over is thus nearly impossible. If no clean-up is applied, significant and unpredictable ionization suppression of different mycotoxins should be expected [123, 124]. Reliable quantification can be achieved only by matrix-matched calibration with model matrix resembling the actual samples as much as possible [124, 184] and also by using isotopically-labeled internal standards whenever possible, preferably one IS per analyte [141, 185, 186]. Structurally analogous internal standards may not compensate for the matrix effect at all, as the ionization efficiency could be different. Although the deuterated IS are preferable, they may still exhibit some unpleasant phenomena, e.g. different extraction recoveries or exchange of deuterium by hydrogen atoms. Stable ^13^C-labeled IS seem to be the best choice for now [186]. In spite of these drawbacks and potential problems, the number of published multi-analyte LC-MS methods for mycotoxins from different chemical groups and their metabolites is increasing and ranges from “small-size” [17, 150, 172, 187] and “medium-size” multi-analyte methods - above 10 different compounds [151, 169, 188] to “large-size” - above 30 compounds in the same chromatographic run [16, 121, 123, 124, 184]. However, the pioneer LC-UV-MS screening method [189] remains unsurpassed by its 474-compound database of mycotoxins and other fungal metabolites, albeit providing only qualitative data.

### 4.2. High performance liquid chromatography with fluorescence detection (HPLC-FL)

Fluorescence detection is by its nature highly specific and sensitive. Several well-established, reliable, robust and sensitive LC methods with fluorescence detection exist especially for the determination of mycotoxins with natural fluorescence, e.g. aflatoxins, ochratoxin A, citrinin [142] and many of them have been established as the official AOAC methods. For the others, pre- or post-column derivatization with suitable reagents has been widely applied, but has been recently superseded by relative simplicity, but comparative specificity of LC-MS methods after the lower cost has made these instruments available to most laboratories. Nevertheless, LC-FL might still be superior in the area of quantitative determination, where the influence of matrix is negligible compared to possible problems with LC-MS quantification [141].

In spite of its specificity for the fluorescing compounds, they have to be well separated on the chromatographic column to enable a reliable quantification. Usually, a reverse-phase stationary phase is used, e.g. C_18_. Base deactivation additionally improves peak shape for polar mycotoxins with carboxylic groups: citrinin, OTA, some fumonisins [138]. Ion-pair HPLC has also been applied for the polar toxins, using tetrabutylammonium hydroxide [138]. Mobile phase should be composed of acidic aqueous phase (acetic acid, trifluoroacetic acid, acidic buffer) to prevent ionization of carboxylic groups, and gradient with methanol or acetonitrile is preferred [138].

Ochratoxin A is probably the mycotoxin most often determined by HPLC-FL. Most authors use the octadecylsilica stationary phase and isocratic mobile phase composed of acetonitrile and water acidified with acetic acid (approx. 1:1) [99, 112, 190–193]. Retention time for OTA is below 15 min under these conditions. Alternatively, a phenylhexyl column is chosen [31]; methanol is sometimes used instead of acetonitrile [194] or a mixture of both [195]. Mobile phase is usually slightly acidified with acids, but pH may not be stable; therefore some authors prefer the use of acidic buffers [195, 196]. Medina *et al.* [134] published a thorough investigation on suitability of various isocratic mixtures for OTA separation and found methanol unsuitable. Peak for OTA was wide and tailing if pH of mobile phase was above 3.5; it was therefore necessary to use phosphoric acid for acidification [134]. Fluorescence is usually measured at λ_ex_ 330–334 nm and λ_em_ 460–464 nm [99, 112, 134, 190–194], but some authors use lower excitation wavelengths 225 nm [195] or 247 nm [196] and different emission wavelength of 480 nm [196]. There is little variation in the chromatographic conditions, but some innovative approaches include automatization of the analytical procedure [197], and inclusion of cyclodextrins [198] or post-column ammoniation [199] to enhance fluorescence. Most publications, however, focus on the sample pretreatment methods to minimize the matrix interferences. Besides the immunoaffinity clean-up [29, 99, 112, 190, 192, 193], conventional SPE is used [31, 112, 194, 196]. Other sample preparation approaches are sometimes applied, such as on-line SPE [118], extract cleaning with lead hydroxyacetate [134], solid-phase microextraction [126, 191] and coacervative extraction [130]. In spite of clean-up, coeluting substances may appear in the chromatograms when dealing with complicated matrix [190]. In such cases, confirmation of OTA identity is accomplished by derivatization to fluorescing methyl-OTA with longer retention times [112, 190, 192, 194] or with LC-MS [194]. Practically all studies apply external calibration or sometimes standard addition method [192], internal standards being rarely used, e.g. diflunisal [200]. Compared to the vast number of articles dealing with HPLC-FL determination of OTA in various matrices, there are only rare publications dealing with similar compounds, e.g. ochratoxin B and metabolite ochratoxin α [201, 202].

Aflatoxins possess natural fluorescence as well, at least AFB2 and AFG2, while AFB1 and AFG1 (see Figure 1) have to be derivatized to enhance their fluorescence [13, 103, 108, 111, 119, 203, 204]. The most usual is derivatization to their hemiacetal form prior to chromatographic separation, using trifluoroacetic acid [13, 103, 108, 119, 203, 204], although post-column derivatization with pyridinium hydrobromide perbromide has been applied as well [111]. Fluorescence detection is accomplished at λ_ex_ 360–370 nm and λ_em_ 418–425 nm [13, 14, 108] or 435–440 nm [103, 111, 119, 203, 205]. The usual chromatographic conditions are reverse-phase (C_18_) column and isocratic mobile phase consisting of 20–50 % organic solvent, usually a mixture of methanol and acetonitrile, and water [13, 103, 108, 119, 203, 205]. Some authors use aqueous phase acidified with phosphoric acid [14, 111]. The determination is frequently performed together with OTA determination. A step-wise gradient is usually applied, switching to isocratic mobile phase suitable for OTA elution after aflatoxins have been eluted (12–20 min) [13, 14, 103, 111, 203]. Considerable accent has been put on sample preparation rather than chromatographic separation, which seems to be non-problematic. Besides immunoaffinity clean-up [14, 29, 103, 108, 111, 203–205] there are some new approaches, e.g. automation of this procedure [111], use of immunoaffinity monolithic discs (CIM) for on-line extraction [105], SPE on silica columns [206] or matrix solid-phase dispersion (MSPD) extraction [119]. Airborne aflatoxins and OTA have been collected in water by passing air through it, followed by immunoaffinity clean-up [14] or sampled on microfibre filter subsequently extracted with organic solvents [13]. Again, there are relatively few methods published for the determination of the metabolite aflatoxin M1 [29, 205].

*Fusarium* mycotoxins, important pollutants in various foodstuffs, do not fluoresce and have to be derivatized, usually before chromatographic separation. With fumonisins, the reaction site is the primary amino group and the reagent most often used is *o*-phthaldialdehyde (OPA) with 2-mercaptoethanol [35, 39, 51, 139, 207–212]. The resulting derivate is highly fluorescing (λ_ex_ 335 nm, λ_em_ 440 nm), but stable only for 4 min [39, 138, 139]. As an alternative to 2-mercaptoethanol as reaction partner for OPA, *N*-acetyl-cystein has been identified as the most suitable [211]. Various other derivatization reagents have been tested: fluorescamine, naphthalene-2,3-dicarboxaldehyde (NDA), 4-fluoro-7-nitrobenzofurazan (NBD-F), dansyl chloride, 9-fluorenylmethyl chloroformate (FMOC), 6-amino-quinolyl *N*-hydroxysuccinimidylcarbamate, fluorescein isothiocyanate (FITC) with different stabilities and sensitivity of detection [39, 138, 139]. NDA derivatives appear to be the most promising alternative [212]. The column for the chromatographic separation of the derivatized fumonisins is reverse-phase (C_18_ or phenylhexyl), but as the compounds still have four carboxylic groups, strongly acidic mobile phase is needed to obtain good peak shape [138]. Usual mixtures are composed of methanol or acetonitrile and acidic buffer at pH approx. 3.5 [35, 207, 208], isocratic or linear gradient. Native fumonisins can be separated on ion-pair column and derivatized post-columnally with OPA and *N*-acetyl-cystein [139]. Immunoaffinity [139, 208, 209, 212] or SPE on C_18_ and SAX columns [139, 207, 210] are applied prior to HPLC. Methods for the metabolites, e.g. aminopentol-1, are seldom published, but use essentially the same procedure [207].

Trichothecenes, another important group of *Fusarium* mycotoxins, are amenable to GC analysis with and without derivatization, therefore this has been the most frequently applied method. The latter is applicable only to less polar compounds, e.g. T-2 toxin, 4,15-diacetoxyscirpenol [136]. However, some methods employing derivatization to fluorescing products and subsequent HPLC-FL analysis have also been published. One of the first was a post-column degradation of DON and nivalenol to formaldehyde by sodium hydroxide, followed by the formation of fluorescent derivate from reaction with acetoacetate and ammonium acetate [39, 136]. Method was later extended to other type-B trichothecenes [39], but is quite experimentally demanding [136]. Coumarin-3-carbonyl chloride is the most often used reagent for off-line derivatization of type-A and B trichothecenes prior to HPLC [209, 213–215]. The derivatives are then separated on reverse-phase columns using a mixture of acetonitrile and water acidified with acetic acid. High percentages of acetonitrile should be present (> 60 %) [209, 213, 215]. Methanol may be used as well [214]. Fluorescence detection is performed at λ_ex_ 292 nm and λ_em_ 425 nm. Excess reagent peaks might interfere with the detection, so it is advisable to perform clean-up after derivatization [213]. Alternative derivatizing reagents include 1-anthroylnitrile, where products are separated under similar conditions as above and detected at λ_ex_ 381 nm and λ_em_ 470 nm [106]; zirconyl nitrate and ethylenediamine, although in this method no HPLC separation was performed [72]; pyrene-1-carbonyl cyanide for T-2 toxin in the presence of cyclodextrins [198]. In spite of these efforts, HPLC-UV [215] and more recently LC-MS [39] are more frequently applied because of relative simplicity and comparable or better sensitivity.

Zearalenone and zearalenols are compounds with native fluorescence. Common HPLC-FL methods thus include separation on C_18_ column with mobile phase with high percentage of organic solvent and detection at λ_ex_ 270–280 nm and λ_em_ 440 nm or 452 nm [39, 45, 93, 116, 209]. Fluorescence can be further enhanced by cyclodextrins [189]. The main emphasis of method development is on the extraction and clean-up of samples before the analysis, mostly using immunoaffinity columns [14, 116, 209]. Alternative approach was accelerated solvent extraction without further clean-up [93]. While HPLC-FL was still the method of choice for zearalenone seven years ago [140], it has now been superseded by LC-MS methods [39, 145].

Citrinin, a mycotoxin produced by different fungal genera, has acidic properties and is in its non-dissociated form a fluorescing compound (λ_ex_ 320–340 nm and λ_em_ 495–512 nm) [19]. Chromatographic separation has been achieved on normal-phase columns, reverse-phase and reverse-phase ion-pair stationary phase [19, 138]. The first method suffers from low retention time reproducibility [19]. In reverse-phase chromatography, mobile phase has to be acidic, while acetonitrile is preferable to methanol to achieve better sensitivity. It is quite difficult to achieve good peak shape for citrinin [19]. Thus, ion-pair RP with tetrabutylammonium hydroxide or phosphate yields the best chromatographic results, but the eluate has to be acidified prior to detection in order to enhance the sensitivity, as the anionic form of citrinin does not fluoresce [19, 138]. Due to the similar chromatographic behavior and fluorescent properties, as well as frequent occurrence in the same type of samples, methods for simultaneous determination of citrinin and ochratoxin A are sometimes encountered [138, 216]. Ergot alkaloids are also sometimes analyzed by HPLC-FL. The published methods are dealing only with determination of ergovaline in various matrices [217–219]. This toxin has the native fluorescence (λ_ex_ 250 nm, λ_em_ 420 nm) and can be separated on reverse-phase column using isocratic mobile phase. Ergotamine may be used as an internal standard [218, 219].

In spite of some obvious advantages of fluorescence detection, such as excellent specificity, low limits of detection, robustness and independence from matrix, HPLC-FL methods have been mostly swept away by LC-MS. The main advantage of the later is in its identification power and possibility to perform determination of multiple analytes from different chemical groups in the same run. Thus, it is expected that the development of methods employing fluorescent detection of mycotoxins has at least temporarily stopped.

### 4.3. High performance liquid chromatography with other types of detection

Compared to the mass spectrometric and fluorescence detection, all other detections available in HPLC are seldom used in mycotoxin analysis. The reasons might be in higher limits of detection unsuitable for trace amounts of the determined substances, and lack of specificity for some of detectors.

A large database of mass spectrometric and UV absorption data for 474 different mycotoxins and other fungal metabolites has been published by Nielsen and Smedsgaard [189]. As can be seen from it, most of the important mycotoxins presented in this review do not absorb in the UV part of spectra at all (e.g. most of trichothecenes, enniatins, fumonisins) or absorb only at rather non-specific wavelengths 200–225 nm (e.g. DON and its derivatives, ochratoxins, some zearalenone analogues) [189].

HPLC methods for the determination of *Fusarium* mycotoxins using UV detection have been published for deoxynivalenol (λ 218 nm) [220–222]; for nivalenol, DON and some of its derivatives [223] or for several type-B trichothecenes (diode-array, λ 221 nm) [215]. In the latter method, UV detection gave better results than FLD of coumarin-3-carbonyl derivatives [215]. Method with simultaneous UV and FL detection of trichothecenes type-B, OTA, zearalenone and citrinin was also developed [216]. Better sensitivity for DON and other type-B trichothecenes can be achieved by electrochemical detection in reductive mode or by derivatization with *p*-nitrobenzoyl chloride and subsequent UV detection at 254 nm [136]. Zearalenone and its metabolites are also amenable to UV detection, e.g. at λ 236 nm [209, 224]. Alternative mode of detection is amperometric [71]. Fumonisins do not absorb UV radiation, but evaporative light scattering detector (ELSD) has been applied in their analysis [138, 225].

Ochratoxin A, routinely analyzed by HPLC-FL, can also be detected with UV (photodiode-array) detector at 333 nm. Its presence was confirmed by peak shift after esterification with BF_3_ [226].

Patulin is a mycotoxin perfectly amenable to HPLC (reverse-phase) with UV detection because of its strong UV absorption - λ_max_ 276 nm [25, 189]. Therefore, some methods have been published [25, 227], but particular care has to be taken to separate patulin from 5-hydroxymethylfurfural, a coeluting compound of non-fungal origin commonly appearing in deteriorating fruit juices [25, 228].

Cyclopiazonic acid exhibits strong UV absorption at cca. 280 nm, therefore some methods for its determination by HPLC-UV (photodiode-array) have been published. Separation and peak shape might be problematic because of the acidic nature of the analyte, but were nevertheless plausible on C_18_ column using a gradient of solvent containing ZnSO_4_ [20]. Another possibility is an aminopropyl-bonded silica as a stationary phase and mobile phase, composed of acetonitrile and ammonium acetate. In this case separation is accomplished by mixed ion-exchange and reverse-phase mechanisms [27, 229].

LC-UV methods for other mycotoxins are not frequently published. Mycophenolic acid in cheese was determined on aminopropyl-silica column with mobile phase at neutral pH and 80 % of organic solvent using a previous extraction with solid-phase microextraction. Detection wavelength was 254 nm [23]. Roquefortine C in cheese was analyzed by on-line extraction and separation on C_18_ column, λ 330 nm [24]. *Alternaria* mycotoxins can be separated on reverse-phase column with UV detection at 240–260 nm [230]. Citrinin exhibits maximal UV absorbance at non-specific 216 nm. Although LC-UV methods exist for its determination, other methods are preferable [19].

### 4.4. Gas chromatography (GC)

Modern gas chromatography combines superior separation on the capillary columns with a variety of general and specific detectors. The obvious disadvantage when compared to LC is the fact that only thermally stable and volatile analytes can be analyzed, although this problem can partially be solved by derivatization. However, as mycotoxins are generally speaking rather large and polar molecules, they are better amenable to LC and GC methods are not common.

Of the published GC methods prevail those developed for trichothecenes and other *Fusarium* toxins [39, 136, 138]. Onji *et al.* [135] report on analysis of native compounds (zearalenone, DON, T-2 toxin and others) using cool on-column injection and there are other reports on direct analysis [136, 138], although adsorption of more polar mycotoxins to column has been observed [136]. An obvious advantage is the combination with MS detection, as the identity of compounds can be confirmed [135], which is especially important in the analysis of metabolites [136]. Direct analysis of T-2 toxin using a GC-GC tandem system for sequential extraction and separation was also accomplished [132]. Most researchers, however, prefer to derivatize mycotoxins to enhance their volatility and decrease polarity. In trichothecenes, hydroxyl groups are usually derivatized to trimethylsilyl (TMS) [136, 231, 232] or trifluoroacetyl (TFA) derivatives [136, 233]. Other reagents yielding pentafluoropropionyl (PFP), heptafluorobutyryl (HFB), trimethylchlorosilyl (TMCS) derivatives and commercial mixtures in various combinations are also used [39, 136, 234]. Introduction of halogens into the molecule renders it detectable by electron capture detector (ECD), which is highly selective and sensitive [39, 136, 223]. Other detectors for any type of derivatives or native compounds include flame ionization detector (FID) [232] and most often mass spectrometer (MS) with electron ionization (EI) [135, 231, 232] or chemical ionization [233]. Fluoroacyl derivatives are amenable to negative chemical ionization (NICI) as well [136]. However, unknown trichothecenes or metabolites are difficult to identify from their MS spectra if they are derivatized [136, 235]. Other *Fusarium* toxins may be derivatized and analyzed by roughly the same procedure, using GC-MS: e.g. fusaproliferin [234], RALs [39, 235]. GC methods for fumonisins have also been published. The protocol included hydrolysis of acidic side chains and their re-esterification or acylation of the remaining aminopolyol. Derivatives were subjected to GC-MS analysis, but none of the approaches was satisfactory in terms of compound identification [39, 138]. Analysis of TMS-fumonisins was also developed more than ten years ago [138], but no further attempts were seen in this direction.

Published methods dealing with GC analysis of other than *Fusarium* mycotoxins are rather scarce. A method for ochratoxin A was developed, employing TMS-TFA derivatization and GC-MS, but it was deemed not suitable for routine quantitation [226]. Some methods for patulin were published, mostly using derivatization to TMS-patulin and ECD or MS detection [25, 236]. In another publication, acylation after diphasic dialysis extraction and GC-MS were proposed [129]. Direct GC-MS of underivatized patulin was possible using on-column injection [25]. Internal standards nitrobenzene [129] or ^13^C-labeled patulin [180] were proposed. Finally, a GC-MS method without previous derivatization was proposed for citrinin [237].

### 4.5. Thin-layer chromatography (TLC) and other chromatographic methods

Thin-layer chromatography (TLC) is a low-cost, rapid analytical technique, yielding qualitative or semi-quantitative estimations by visual inspection, but with densitometric measurements also reliable quantitative results [39, 69, 145]. These methods were mostly developed before the great development and affordability of HPLC and LC-MS instruments and some were established as the official AOAC methods. Their many advantages include suitability for crude extract analysis, a wide choice of stationary and mobile phases, as well as an array of spraying agents used for the detection [69]. In spite of that, TLC methods are now rarely used for other than screening purposes. Nevertheless, there are some more recent publications showing the interesting possibilities offered by this technique.

A TLC method with previous immunoaffinity clean-up and densitometric quantification has been developed for aflatoxins in food samples. Advantages of the method include fast sample processing and limits of quantification lower than established by regulatory organs [238]. Overpressured-layer chromatography [239], two-dimensional TLC and high performance TLC (HPTLC) are other possibilities for efficient separation and determination of aflatoxins [240]. Sterigmatocystin, a precursor to aflatoxins, has been determined on amino-derivatized HPTLC plate and reagent-free (only heating) densitometric detection [241].

While aflatoxins are naturally fluorescent compounds and their detection in TLC is not problematic, trichothecenes require post-development visualization with spraying reagents: *p*-anisaldehyde, 4-*p*-nitrobenzylpyridine [39], and aluminum chloride for DON [242]. Recent methods include quantitative determination of DON and T-2 toxin in cereals by high performance TLC [242, 243]. Fumonisins have also been determined in corn using reverse-phase TLC plate, spraying with fluorescamine in borate buffer/acetonitrile mixture, and densitometric detection [244]. In contrast to older methods [39], this application has had acceptable limits of detection for routine analysis [244]. In another method, fumonisin B1 has been analyzed by TLC after clean-up on ion-exchange column. The spraying reagent was *p*-anisaldehyde, detection was by laser-scanning densitometry [245]. Zearalenone emits fluorescent light upon excitation. Quantitative methods are based on TLC with densitometry [246] or HPTLC [247]. Moniliformin, a low molecular weight *Fusarium* mycotoxin, can also be determined by TLC and visualized by 3-methyl-2-benzothiazolinone hydrazone (MBTH) or 2,4-dinitrophenylhydrazine with low limits of detection [39].

TLC determination of ochratoxin A (native fluorescence-densitometric detection) was compared to different HPLC methods. Recoveries for the TLC method were lower than average of HPLC methods [99]. There hasn’t been much development in recent years for the TLC methods for patulin. Generally, the analyte has to be visualized by MBTH or ammonia fumes. Separation from the commonly present contaminant in apple juice, 5-hydroxymethylfurfural, was often problematic [25]. The situation with citrinin is similar, as the TLC methods used before the expansion of HPLC-based protocols were generally not sensitive enough. Citrinin was visualized by exposure to aluminum chloride, pyridine or acetic anhydride. Silica plates had to be impregnated with acid to achieve symmetrical spots [19].

Other chromatographic techniques besides HPLC, GC and TLC are a rarity in mycotoxin analysis. Moniliformin from maize plants was separated by hydrophilic interaction chromatography (HILIC). Detection was accomplished by UV detector at 229 nm and with negative-mode electrospray mass spectrometer (ESI(−)-MS) [248]. Combination stationary phases operating both through RP-hydrophobic and hydrophilic interactions are expected to find broader application in the future due to good retention of neutral, acidic and basic compounds. They are especially well suited for multi-toxin LC-MS/MS methods where a great number of chemically diverse mycotoxins should be separated in a single chromatographic run [249]. Trichothecene analyses in fungal cultures were performed by supercritical fluid chromatography (SFC) and electron ionization MS detection (EI MS) in the heyday of this technique, but there were no further developments [136]. High-speed counter-current chromatography (HSCCC) has been used as a preparative rather than analytical technique for isolation of altertoxin I from *Alternaria* species [250] or some novel fungal compounds from *Penicillium* species [251].

### 4.6. Capillary electrophoretic (CE) methods

In spite of the great separation power and versatility of capillary electrophoretic (CE) techniques, they have never gained such popularity as HPLC, although the same analytes can be determined with CE and the same detectors can be used. Possible explanation might be in better detection limits and greater user-friendliness of HPLC methods.

Achieving low enough detection limits might present a serious problem in CE, therefore the mycotoxins for which CE methods have been developed are mostly those for which fluorescence detection (FL) can be used. Ochratoxin A was determined by CE-laser induced fluorescence (LIF) in human serum [55], food samples after immunoaffinity clean-up [252], and in wine after combined extraction with SPE and supported liquid membrane [131]. Capillary zone electrophoresis (CZE) coupled to LIF was applied to OTA analysis in house dust [253] and human serum [254]. Aflatoxins in animal feed were determined by micellar electrokinetic capillary chromatography and FL [70]. Cyclodextrins were applied to enhance native zearalenone fluorescence in CE-LIF analysis [255], and for the same purpose in CE-LIF determination of T-2 toxin, which was previously derivatized with pyrene-1-carbonyl cyanide [198]. Another detector commonly used in CE is UV-spectrometric. Methods have been developed for determination of patulin in apple juice, employing micellar electrokinetic capillary chromatography (MEKC) [256] or microemulsion electrokinetic chromatography (MEEKC) [257] and UV detection at 276 nm. Limits of detection were in the μg/L range, which is satisfactory for routine analysis. In contrast to the CE-FL methods for ochratoxin A mentioned above, UV detection of OTA after CE separation has given quantification limits that were too high for the analysis of real samples [195].

### 4.7. Other analytical methods

Applications of analytical methods other than separation or immunoaffinity-based techniques, e.g. to study the electrochemical behavior of mycotoxins [258], are sometimes encountered, although the applied concentrations are too high to be applicable to trace analysis. The only other technique used in mycotoxin analysis in the food samples is ion mobility spectrometry (IMS). It has been applied to determination of aflatoxins in pistachios, the ionization method was corona discharge [259]. Another example is the determination of zearalenone in maize samples by high-field asymmetric waveform ion mobility spectrometry - mass spectrometry (FAIMS-MS) with electrospray ionization [260]. Both methods achieved good limits of detection.

## 5. Determination of mycotoxins in various matrices

After the general overview of the screening, sample preparation, clean-up and analytical techniques, more detail on the published methods for the individual mycotoxins in various matrices is presented in Table 2.

The main considerations when choosing the appropriate set of techniques suitable for the mycotoxin and the sample are the following: homogeneity of the sample is achieved with greater ease with liquid samples, to which also direct solid-phase extraction techniques may apply. For solid samples, however, the sample comminution and homogenization is the most crucial step to assure the representative sample. Extraction is then almost invariably performed by solvents, using either classical (Soxhlet) or novel approaches (PLE, SFE, MAE). Decision to clean-up the crude extract depends mainly on the type of matrix and presence of potentially interfering compounds, e.g. sugars, fatty acids etc., as well as detection mode. Finally, the choice of the analytical method depends mainly on the chemical characteristics of the mycotoxins.

## 6. Pitfalls of mycotoxin analysis

Mycotoxin analysis presents several problems for the analytical chemistry, many of which have already been at least in part addressed in the previous sections. There is a formidable diversity of chemical structures and associated physico-chemical properties, and on the other hand a vast array of samples containing only trace amounts of these compounds, but a lot more of interfering substances. As we are dealing with an opponent that has to be respected, we feel it is necessary to reemphasize and elaborate on certain aspects of mycotoxin analysis that are exceedingly important for the final result. The accuracy of the latter is, as ever, dependent on the accuracy of the steps taken to obtain it [283].

### 6.1. Metabolites and other transformation products

Although grouped in the same subsection, there are several separate problems that have to be addressed here. The first question to be asked is: do we really determine all of the mycotoxins present in our sample by the chosen analytical method? Apart from the loss of the analytes during the sample preparation step and inaccurate analytical determination, which will be discussed later, the problem frequently arises from the fact that we are looking only for the free mycotoxins in the sample and forget about their various conjugates, which nevertheless cause the same or similar physiological impact when ingested. Conjugated mycotoxins result from the infected plant metabolism of the mycotoxins excreted by the infecting fungi [16, 158]. Our knowledge of the mycotoxin identity stems mainly from their isolation from the laboratory-grown fungal cultures [189], but metabolites formed in infected plants have been taken in consideration only recently and nicknamed “masked mycotoxins” [16, 158]. As an example, at least 17 different metabolites/conjugates of zearalenone have been identified in the model plant [158]. Detection and identification of novel metabolites in a complex mixture is a hard task, therefore protocols and computer algorithms are developed to aid in this process [284]. Important conjugates in terms of frequent occurrence have been found for zearalenone [16, 157], ochratoxin A, and DON in wheat and maize [16]. Processing of foodstuffs, e.g. cooking and frying, is another source of mycotoxin conjugation [16] or transformation [9, 19, 285]. Fumonisin B1 was thus shown to react with sugars, amino acids and proteins during cooking [16], while citrinin was transformed into more toxic product citrinin H1 [19] and less toxic citrinin H2 [19, 285], depending on the temperature of heating. Finally, mycotoxins can be degraded upon exposure to light, yielding products of unknown toxicity. This has been shown for roquefortine C in cheese [24] and for ochratoxin A in wines [118].

Determination of mycotoxins in food samples derived from animals (meat, dairy products, eggs) presents other challenges. Some mycotoxins exhibit very low carry-over from feed to animal products: DON, fumonisins, zearalenone [3, 282], e.g. the transmission rate of DON to eggs was shown to be 15–29·10^3^:1 [274]. Nevertheless, some metabolites have been identified, e.g. deepoxy-DON in pig urine [149] or aminopentol-1, a fumonisin B1 metabolite, in swine liver [207]. Zearalenone is metabolized primarily to α-zearalenol and β-zearalenol, but further into zeranol and taleranol [155]. Subsequent metabolites include also α-zearalanol, β-zearalanol and possibly zearalanone [109, 286], although the latter couldn’t be detected in all studies [155]. Things get further complicated with zearalanone, because it has been used (and still is) as a very satisfactory internal standard in the mass spectrometric determination of zearalenone and its first metabolites [153, 154] before it was known that it occurs in nature as well. Finally, a banned anabolic substance for farm animals zeranol also yields metabolites taleranol, zearalanone and zearalenols [160].

Ergovaline, an *Ergot* mycotoxin, was shown to be excreted into milk only in a limited amount [219], similarly to ochratoxin A, which accumulates mainly in blood, liver and kidney, but not in muscles [3]. However, OTA is excreted in urine as a conjugate with glucuronic acid [287].

One of the most important metabolites to be determined in animal samples is aflatoxin M1, occurring in milk and dairy products [29, 63, 205, 276–278]. Carry-over of aflatoxins has been estimated to 1–2 % [3]. Unchanged aflatoxin B1 was excreted in eggs at transmission rate 5000:1 [275]. In meat and meat products, other metabolites besides M1 have been detected: aflatoxin M2 and P1, aflatoxicol [108].

Besides food and feed, other samples are of importance in mycotoxin analysis. Environmental samples are a potential source of contamination of plants and drinking water, but degradation products of mycotoxins may occur as well. In the survey of various real-life aqueous samples for zearalenone, it was present in drainage water from the infested field, but none of its metabolites were detected [156]. In another research, degradation of zearalenone and OTA was studied in agricultural soils. Half-lives were about 10 days for ZON and below 1 day for OTA, but no degradation products were identified [32]. Human biological fluids (blood, urine) are monitored for the presence of mycotoxins and their metabolites for the purpose of assessing and estimating the exposure of population [283]. While native compounds are most frequently determined, metabolites - if known - can usually be better biomarkers of exposure [279, 283, 287].

### 6.2. Choice of the representative sample

Sampling is an extremely important part of the overall analytical procedure in general, but even more so in the determination of mycotoxins. The reason is a highly heterogeneous distribution of analytes (“hot spots”) in the lot of food/feed commodities. Moreover, the sampling stage of the analysis directly influences the costs of the control procedure [283]. In spite of the extreme importance of sampling, the laboratory performing the analysis usually has no influence on it [39]. It has been estimated that the coefficient of variation (CV) in the determination of aflatoxin concentration in peanuts is between 60 % and 120 % for sampling, and only up to 20 % for subsampling and up to 10 % for the analysis [283, 288]. Interestingly, DON determination in wheat showed lower CVs for sampling than for sample preparation in spite of small sample size (< 0.5 kg) [289]. It would therefore seem that sampling error might depend both on the type of sample and mycotoxin. The effects of improper sampling and subsequent inaccurate determination are either that unsuitable lots are put on the market (false negatives: risk for the consumers) or harmless lots withdrawn (false positives), presenting a financial burden for the producers [283, 288].

Standardization of sampling procedures is therefore absolutely necessary to minimize the occurrence of both false positives and negatives [288]. On the other hand, the sampling procedure should also be fast, cost-effective and easy to apply [290]. Although most samples needed to be analyzed are from the food/feed lots waiting to be released on the market, sampling should not be forgotten elsewhere, e.g. in the research of mycotoxin contamination of grain from field to the consumer, where proper sampling procedures in each step also affect the final result [291]. Some strategies to decrease the determination error associated with the sampling are reviewed below.

The simplest method to reduce sampling error is by increasing the sample size [288], although this may be impractical. Another approach is to locate a considerable number of sites (>100) in the lot and form an aggregate representative sample from these partial samples [283]. This approach, however, requires a statistically correct sampling plan to cover the whole lot [290]. Another problem with big samples is their subsequent homogenization and comminution in order to obtain a representative laboratory subsample. It has been shown that slurry mixing of samples results in lower CVs than dry milling [292], although the obtained slurry presents problems for the disposal after the analysis [290].

Some novel methods aim to avoid these difficulties by performing a more-or-less “sampling-free” screening. Lots deemed positive should be sampled and analyzed to confirm the result, but the proposed approach nevertheless considerably decreases time and costs.

An automated sampling system DiscoveryCERT FQS™ developed for the extraction and collection of chemical hazard contaminants from cargo pallets has been implemented to the sampling of corn, pistachio and wheat samples for the mycotoxin analysis. Samples of up to 50 kg could be loaded in the system [290]. The system uses pressure cycling, vibration, air jetting, air blasts etc. for the agitation and release of vapor and particles from the sample material, which are then collected on glass fiber filters with a sampling lance. The filter materials are ground or made into aqueous slurry for subsequent analysis. The results correlate rather well with the concentration of mycotoxins determined by the standard sampling approach [290].

Electronic noses detecting volatile compounds produced by fungi have been applied not only to detect fungal contamination of grain [84, 85, 87] and apples [86], but also to predict mycotoxin concentration [86, 87]. Similarly, solid-phase microextraction or headspace withdrawal with subsequent GC-MS analysis can be used for the same purpose [82, 84–87]. These methods can be performed on the whole lot (or rather over it) of food/feed commodity.

As regards subsampling, the usual procedure is mixing and grinding of the sample to ensure homogenization. However, it has been shown that the distribution of the mycotoxin contamination is related to particle size [77, 283]. Grinding procedures yielding finer particles, such as slurry mixing [292], are therefore preferable, as well as analysis of the most representative ground fraction separated by sieving [77]. Larger subsamples are also associated with lower CVs [283].

### 6.3. Sample preparation bias

The next step in the analytical determination of mycotoxins is the preparation of the samples for the subsequent analysis, which is also prone to various errors. One dubious advantage may be that this step is usually performed by highly trained laboratory staff, which can more readily observe the possible problems. The well known problems with sample preparation are insufficient purification and consequent interferences during the analysis; introduction of artifacts because of too harsh extraction conditions (e.g. in Soxhlet apparatus); loss of analyte and incorrect estimation of its recovery. A good approach to avoid especially the last problem is the introduction of internal standard in the sample before the extraction and clean-up. Other critical points of sample preparation are highlighted below.

Generally, sample preparation consists of extraction and clean-up step, which may be performed together in some approaches, e.g. if the extraction is selective enough. On the other hand, the raw extract (i.e. without purification) can already yield satisfactory results in immunoassays [39, 57]. Several publications have shown that the analysis of raw extracts can be successfully accomplished by LC-MS [123, 124, 142, 152]. In spite of these reports, coextracted matrix compounds may present a problem even for selective MS detection. Even in the case of good chromatographic separation from interferences, which are “visible” only in one-stage mass analysis, there remains a greater chance of pronounced matrix effects - ionization suppression in LC-MS interface with raw extracts, leading to erroneous quantification [91, 142, 146]. Use of internal standards is therefore practically mandatory in such experiments [39, 142, 146], but even this may not always suffice [174, 186].

Raw extracts are usually obtained by solvent extraction of the sample material. The choice of the solvent or a mixture of solvents is of great importance here, having a direct impact on the recovery of mycotoxins from the matrix. Although the mixture of acetonitrile or methanol with water is most frequently applied, it is not equally suitable for all mycotoxins from every matrix [142, 224, 267]. Especially mycotoxins with acidic functional groups (fumonisins, ochratoxin A) require an addition of acidic modifier to enhance the recoveries, while some samples, e.g. animal tissues, respond better to polar organic solvents [39, 142]. Various substances are added to the sample to precipitate bulk matrix components [134] or enhance mycotoxin extraction [142]. To speed up the extraction step, several approaches have been devised: shaking, blending, microwave assisted extraction (MAE) [97], pressurized liquid extraction/accelerated solvent extraction [39, 142], but especially in the latter higher levels of coextracted matrix compounds have been observed [142]. Additional critical steps are phase separation [267] and defatting with partitioning into nonpolar organic solvent, which is needed for extracts from samples with high lipid content [142]. Both may lead to further analyte loss. Another consideration is about the conjugated mycotoxins in some matrices, e.g. urine, which are better off hydrolyzed prior to extraction [142]. Finally, a word of caution regarding the estimation of recoveries and method calibration using the spiked samples. As it has been shown for many matrices, mycotoxins added to the sample are not so strongly embedded in the matrix as the native ones, therefore longer extraction times are needed for the naturally contaminated samples [39, 138].

The most frequently used approach for the clean-up of raw extracts is the use of immunoaffinity columns (IAC), which show high selectivity for chemically similar group of mycotoxins [39, 142, 146] or to a certain extent even for single compounds [38]. If single compounds are the target, cross-reactivity of IA material may present a serious problem with non-specific analytical method [36]. The purification of extracts by IAC for chemically similar group is very efficient and is thus especially suitable in combination with non-specific analytical techniques, but is considered by many researchers an analytical “overkill” if used prior to highly selective LC-MS/MS analysis [142]. In the era of multitoxin methods performed by LC-MS/MS [16, 121, 123, 124, 184], compound-specific purification of extracts even presents an obstacle [142]. Other drawbacks of IAC include rather low capacity [224], significant loss of some analytes (e.g. fumonisins) [264] and incompatibility of columns with some matrices [265]. The comparably higher costs of IA columns should also be considered.

Moleculary imprinted polymers (MIPs) are increasingly popular as an alternative to IAC for selective clean-up of extracts [65, 66]. While being cheaper, reusable and less sensitive to harsh conditions than IAC, they have problems of their own: non-specific binding, lower reproducibility, potential contamination of the extract by template bleeding [68]. The commercial availability is currently low, although this is expected to change in the future [65, 293].

Conventional solid-phase extraction (SPE) is the mainstay of extract purification for mycotoxin analysis, especially the MycoSep line of multifunctional cartridges, designed for easy and rapid handling [39]. However, they have been shown to have low recoveries for zearalenone [142]. A variety of SPE cartridges packed with a single sorbent are used as well: strong anion-exchange resins (SAX), aminopropyl, carbon materials, polymeric resins, reverse-phase (C_18_) etc. The choice of sorbent depends on the nature of the analyte and the sample, but also on the analytical method used [39, 112, 142]. As an example: SAX cartridges are especially suitable for acidic mycotoxins, e.g. fumonisins, but their hydrolyzed metabolites are not retained [39]. On the other hand, RP sorbent material is rather unspecific, but is thus advantageous in combination with multitoxin LC-MS/MS analyses [142, 146]. One additional advantage of SPE is its ease of automation [58]. Some extraction methods are sufficiently specific and the extracts don’t need further clean-up. One example is the supercritical fluid extraction (SFE) [39, 98, 142], but unfortunately this approach is quite expensive and is thus not expected to gain broad acceptance. Solid-phase microextraction (SPME) has been applied to extract some mycotoxins from solid samples without any further purification [23, 27, 126].

There are screening methods that don’t require any sample preparation, e.g. certain immunoaffinity assays [41], electronic noses [84–87] and IR-based methods [76, 77, 80]. They may have an advantage of circumventing this stage and its associated errors, but are instead prone to their own, which have been covered in detail in the appropriate section of this review. Moreover, the unseparated matrix compounds can greatly affect the results, especially in immunoassay methods [43].

### 6.4. Analytical method bias

Analytical method, broadly speaking, encompasses also the sampling and sample preparation steps, but in this section the focus will be only on the final analytical determination of mycotoxins in the purified extract.

The particular problems connected with the individual techniques have been addressed in detail previously in the appropriate sections of this review, here we provide only a short overview. Many screening methods, but especially immunoaffinity assays, in particular broadly used ELISA, should be used with caution, as they tend to produce false positives or yield too high values due to cross-reactivity and matrix interferences [35]. Interferences of course present the problem also in the more definitive techniques: GC, HPLC. Whenever derivatization of analytes is needed prior to detection, the recovery of the reaction should be closely monitored, and the stability of derivatives might also be a serious problem [39, 138, 211]. With LC-MS or LC-MS/MS methods, ionization suppression in the interface due to visible and “invisible” (i.e. coeluting over a broad range of retention times) matrix interferences is a well-recognized problem, sometimes observed also in GC-MS methods [39, 123, 142, 146, 151, 174, 234]. The proposed solutions include extract dilution [142], matrix-matched calibration [124, 150, 174, 234] and use of internal standards [151], preferably isotopically labeled [142, 146, 159, 176, 180, 186]. In multitoxin methods, more than one internal standard should be added [142] - in fact, the best results are expected when using a corresponding isotopically-labeled IS for each analyte!

All methods for the analysis of mycotoxins should be fully validated by establishing compliance to various criteria: selectivity, linearity, limit of detection, limit of quantification, decision limit, detection capability, intra- and interday precision, recovery, accuracy, robustness etc. [142, 294]. However, this may present additional complications. The first problem is the lack of the calibrants, i.e. sufficiently purified mycotoxin standards. One common problem with the preparation of analytically pure standards is the assessment of their purity [295, 296]. As was shown in the previous sections, different mycotoxins are to a variable extent amenable to various types of detection, while the impurities are completely unpredictable. The search continues for a universal, analyte-independent detector that would have the same response for standard compound and the impurities. Evaporative light-scattering detector has been proposed as such [138], although it doesn’t meet all criteria. Other types of “universal” detectors showing promise are pulsed-discharge photoionization detector and charged aerosol detector (CAD). Another approach is the combined analysis of prepared calibrants by very different methods, e.g. NMR, GC-MS, LC-MS, differential scanning calorimetry etc. [295], or by intercomparison studies in which several experienced laboratories participate [297].

Even with available mycotoxin standards, matrix-matched calibration, which is absolutely necessary [124, 150, 174, 234], may present problems such as interaction between added standard compound and components of the matrix, causing decreasing recoveries [263]. Therefore, the golden standard of validation, certified reference materials (CRMs) should be used in the process of method validation, but not many of them are available at the moment [39, 277, 294, 296, 297]. Interlaboratory studies and comparisons also help to reveal potential methodological problems with mycotoxin analysis in individual laboratories [39, 273, 296, 297].

## 7. Conclusions

The review presented here covers an enormous subject of various aspects of mycotoxin analysis, which can be readily estimated from the number of references and considering that only the interval from 1998 to present has been covered, but by no means exhaustively. Thus, we hope that we have given an overview of the current status of analytical methods for mycotoxin determination in the broadest sense, and also hinted at the trends that might be promising and take over in the future. Various problems of the mycotoxin analysis have also been tackled. However, this review can’t afford to go into too much depth due to the broadness of the subject; the interested reader is therefore directed to the other excellent reviews that focus more on certain groups of mycotoxins [16, 17, 19, 25, 26, 39, 41, 98, 136–140, 262, 266, 293] or the applied analytical methods [12, 17, 34, 41, 52, 59, 69, 136, 142–145, 189, 296].

## Figures and Tables

**Table 1.** Major mycotoxin groups or individual compounds, the fungal species producing them and health effects they cause. **S**ymbols for mycotoxins are written in {} curly brackets. *NUA* denotes “not unequivocally established”.

Mycotoxin(s)	Fungal species	Major health effects
Aflatoxins {AFs}: B1, B2, G1, G2	*Aspergillus* ssp.	hepatotoxic, immunosupressive, carcinogenic, teratogenic, mutagenic
Sterigmatocystin {STC}	*Aspergillus* ssp.	precursor to aflatoxins
Fumonisins {FUMs}: B1, B2, B3	*Fusarium* ssp.	liver and kidney tumors, oesophagal cancer, lung oedema (swine), leukoencephalomalacia (horses)
Trichothecenes - type A: T-2 and HT-2 toxin, neosolaniol {NEOS}, diacetoxyscirpenol {DAS}	*Fusarium* ssp.	weight loss, diarrhea, dermal necrosis (poultry)
Trichothecenes - type B: deoxynivalenol or vomitoxin {DON}, nivalenol {NIV}	*Fusarium* ssp.	food refusal and vomiting, kidney problems, immunosupression (swine)
Resorcyclic acid lactones {RALs}: zearalenone {ZON}, zearalanone {ZAN} α- and β-zearalenol {ZOL}	*Fusarium* ssp.	oestrogenic effects, reproductive toxicity
Ochratoxins {OTs}: A, B, α	*Aspergillus* and *Penicillium* ssp.	kidney and liver toxin, carcinogen; chronic toxicity as accumulates in body
Ergot alkaloids: ergovaline, clavine alkaloids, lysergic acid derivatives and others	*Claviceps*, *Neotyphodium*, *Epichloe* ssp. *Fusarium*, *Beauveria*,	ergotism: gangrene, central nervous system symptoms (convulsions), gastrointestinal symptoms (vomiting)
Enniatins: A, A1, B, B1, beauvericin {BEA}	*Halosarpheia*, *Paecilomyces*, *Polyporus*, *Verticillium* ssp.	acutely toxic, cardiac symptoms, herbicidal, insecticidal, antibiotic
*Alternaria* toxins: altertoxins {ATXs}, alternariol {AOH}, altenuene {ALT}, radicinin and others	*Alternaria* ssp.	acute toxicity (*NUA*)
Patulin {PAT}	*Penicillium* and *Aspergillus* ssp.	acutely toxic (*NUA*), genotoxic, carcinogenic, teratogenic, antibiotic
Moniliformin	*Fusarium* ssp. *Penicillium*, *Aspergillus*,	acutely toxic, cardiac impairment
Citrinin {CIT}	*Monascum* ssp.	hepatonephrotoxic, antifungal, antibiotic
Cyclopiazonic acid {CPA}	*Aspergillus* and *Penicillium* ssp.	weight loss, nausea, diarrhea, giddiness, muscle necrosis, convulsions
Roquefortin C {RQ} Mycophenolic acid {MPA}	*Penicillium roqueforti*	RQ: acutely toxic (*NUA*), neurotoxic MPA: immunosupression, mutagenic, antibiotic

**Table 2.** Methodology for determination of mycotoxins in different matrices.

Matrix	Mycotoxin Type	Methodology		References
		Sample Preparation	Instrumentation	
*Beverages*				
Alcoholic drinks (must, wine, beer)	OTA, DON, AFs	LLE (PEG) SPE (IAC, MIP, on-line) SPME biosensors	HPLC (FD, DAD, MS/MS) GC (MS) CE (DAD)	[56] [61] [68] [101] [110] [112] [118] [125] [130] [131] [133] [134] [173] [191] [193] [195] [196] [197] [199] [226] [261] [262]
Juices (e.g. apple juice)	PAT, AOH	LLE SPE ELISA Dialysis extraction	HPLC (UV, DAD, MS, MS/MS) MEEKC MEKC GC (MS) MS	[46] [86] [129] [178] [179] [181] [227] [228] [236] [256] [258] [263]
*Food and feed matrices*				
Cereals	FUMs (B1), FUS, STC, CIT, Trichotecenes (A, B), T2, HT-2, OTA, RALs, ALT, volatile metabolites	LLE SPE (IAC) Stable isotope dilution ASE Immunosensor SFE HS biosensors	HPLC (FD, MS, MS/MS) GC (MS, ECD) derivatization SFC NIR+PCA HPTLC	[61] [80] [84] [92] [93] [98] [106] [124] [135] [136] [151] [159] [177] [182] [194] [213] [215] [222] [231] [233] [242] [258] [264] [265] [266]
Maize (corn)	AFs, moniliformin, FUMs, FUS, DON, RALs, Trichothecenes (A, B, D), BEA, CPA, CIT	LLE SPE (IAC, on-line) MSPD SFE Immuno-ultrafiltration ELISA Immunosensor OPLC	HPLC (UV, DAD, FD, MS, MS/MS, ELSD) derivatization TLC GC (ECD) Amperometric detection CE (LIF) FAIMS-MS HILIC NIR PCR MED IC	[43] [50] [55] [71] [76] [77] [89] [96] [108] [120] [128] [139] [147] [149] [150] [162] [164] [165] [167] [168] [169] [210] [212] [213] [234] [239] [245] [248] [258] [267]
Wheat	Trichothecenes, AFs, RALs, FUMs, volatile metabolites	LLE SPE (IAC) ELISA SPME MAE	LC (MS) GC (FID, ECD) FTIR+PCA	[35] [60] [75] [84] [97] [216] [221]
Rice	AFs, FUMs, CIT, OTA, Trichothecenes B, FUMs, FUS-X, STC, ZON	SPE ASE	HPLC (UV, FD; MS, MS/MS) GC (ECD, MS) HPTLC	[94] [243] [268] [269]
Soy	Trichothecenes, ZON		HPLC (UV) GC (MS)	[270]
Peanuts, tree nuts, pistachios, seeds	AFs, AOH	LLE SFE ELISA MSPD	HPLC (FD, MS) IMS	[95] [119] [122] [259]
Coffee	OTA	LLE SPE (IAC) SPME	HPLC (FD) CE (LIF)	[99] [190] [252]
Cocoa products, chocholate	AFs, CPA, OTA	SPE (IAC)	HPLC	[271] [272] [273]
Herbs, spices	AFB1, OTA	ELISA		[54], [57]
Red paprika	AFs, OTA	LLE SPE (IAC)	HPLC (FD) NIR	[81], [203]
Bee pollen	OTA, AFs (B1, B2, G1, G2)	IAC	HPLC (FD	[103]
Eggs	AFs (B1), DON, ZON	IAC	HPLC (MS)	[274] [275]
Fish	RALs	On-line MSPD SPE	HPLC (MS/MS)	[28]
Meat, meat products	AFs	IAC	HPLC (FD)	[108]
Milk, dairy products	AFs (M1), OTA, ergovaline, DON, ZON, FUMs, T-2, MPA, RQ, CPA	LLE SPE (IAC) ELISA	HPLC (FD, MS/MS) Amperometric detection	[23] [24] [27] [29] [63] [183] [187] [201] [205] [219] [276] [277] [278]
*Biological samples*				[142]
Urine, plasma	Trichothecenes, FUMs (B1), OTA, RALs, taleranol, RQ	SPE (IAC) SPME	HPLC (MS, MS/MS)	[30] [102] [109] [127] [149] [155] [160] [161] [218] [279] [280]
Blood	OTs (A, B), MPA	LLE ELISA	HPLC (FD, MS/MS) CZE (LIF)	[202] [254] [281]
Liver, kidney, lung, brain,…	FUMs (B1), ZON, AFs	SPE	HPLC (FD)	[207] [282]
*Environmental samples*				
Water	AFs (B1), DON, OTA, RALs	SPE (IAC, MIP, RAM) biosensors	HPLC (FD, MS, MS/MS) GC (MS)	[33] [156]
Soil	RALs, OTA	LLE SPE Soxlet extraction	HPLC (FD)	[31] [32] [91]
*Indoor environment*				[171]
House dust	Trichothecenes, OTA, STC	LLE SPE	CE TLC HPLC (MS/MS)	[170] [253]
Building material	AFs, STC, CIT, OTA Volatile metabolites	LLE SPE	HPLC (MS/MS)	[11] [12] [172]
Indoor air	OTA, AFs, ZON	LLE	HPLC (FD)	[13] [14] [148]
